# Ventral striatum temporal interference brain stimulation enhances the reward positivity event-related potential and reduces anxiety

**DOI:** 10.21203/rs.3.rs-8767480/v1

**Published:** 2026-02-09

**Authors:** Jonathan S. Ryan, Timothy J. McDermott, Boris Botzanowski, Jessica R. Kubert, Samantha A. Betters, Aje Oluwagbohunmi, Summer B. Frandsen, Travis M. Fulton, Adam Williamson, Michael T. Treadway, Negar Fani

**Affiliations:** Emory University; Emory University; Emory University; Emory University; Emory University; Emory University; Emory University; Emory University; St. Anne’s University Hospital; Emory University; Emory University

**Keywords:** temporal interference, striatum, electroencephalography, reward, anxiety, deep brain stimulation

## Abstract

**Background:**

Temporal interference (TI) electrical stimulation is a novel neuromodulation method that can target deep brain structures to enhance the impact of behavioral techniques. We tested the feasibility of TI in targeting the ventral striatum (VS) in concert with an emotion regulation practice (breath-focused mindfulness meditation). As an assay of target engagement, we used electroencephalography (EEG) to measure amplitude of the reward positivity potential (RewP), an event-related potential ERP associated with VS function.

**Methods:**

Nineteen healthy adults (age range: 18–36 years) received an MRI scan and two stimulation sessions during meditation: one active 8 Hz TI stimulation and one 0 Hz sham TI. Before and after meditation, they completed a reward task during EEG recording.

**Results:**

A significant time by stimulation type effect was observed with the RewP win-loss difference (*p*<.001; *η*_*p*_^*2*^ = .61), but not an unrelated ERP, the Cue-N200 (*p*=.93). Active TI, but not sham, led to a RewP increase to win trials from pre- to post-stimulation, with evidence of a sustained RewP win amplitude increase at visit 2 in participants who received active TI at visit 1 (*p* < .001). Decreased negative emotionality was observed for active TI; emotion changes corresponded with increased RewP amplitude to win trials for only active TI.

**Conclusion:**

Findings show support for TI targeting of VS in conjunction with meditation to elicit self-reported and neural change in emotion-regulation and reward processing. Although replication is warranted, our data indicate the precision and short-term continued effects of TI on the VS and reward-related functioning.

## 1. Introduction

Temporal interference (TI) brain stimulation is a relatively new, non-invasive neuromodulation technique that enables targeted stimulation of deep brain regions with fewer off-target effects than conventional non-invasive neuromodulation (e.g., transcranial alternating current stimulation, tACS)(1). The application of TI involves two (or more) high-frequency electrical currents offset by a small frequency difference (e.g., 5000 Hz and 5008 Hz) delivered through scalp electrodes; where these fields overlap in the brain, they interfere to produce an amplitude-modulated electric field whose envelope oscillates at a beat frequency (e.g., 5–8 Hz)(2). Although neurons typically do not respond to high-frequency fields(3), the resulting envelope from their interference can effectively modulate neural activity like a low-frequency signal, with fewer effects on non-target regions(1). Evidence for this specificity comes from a growing number of studies wherein TI has been used to causally manipulate activity in deep brain regions to enhance psychological function in humans, such as hippocampus activity and memory performance(4), or ventral striatum and motor learning(5, 6). Given its ability to directly modulate deep-brain function, TI holds promise both as a mechanistic tool for probing neural circuitry and as a potential therapeutic intervention for neurological and psychiatric disorders, the pathophysiology of which often involve subcortical structures(7).

The ventral striatum (VS) is a region centrally involved in reward processing(8), indicated in both human neuroimaging studies(9) and translational rodent models(10–12). Blunted striatal responses to reward have emerged as a potential neural marker of anhedonia—the diminished ability to experience reward or pleasure—which is a core symptom of depression(13, 14) among other disorders(e.g., schizophrenia). Recent meta-analyses suggest that adults with depression exhibit reduced VS activation in response to rewarding stimuli as compared to healthy adults(15, 16), and those with reduced VS responses have shown worse long-term symptom outcomes(17). Deep VS stimulation for severe depression has shown promise in alleviating symptoms(18), but its cost and invasiveness limit feasibility for widespread clinical use. Targeting the VS with TI at a putatively excitatory frequency (e.g., 8 Hz;(19)) offers a non-invasive alternative to directly manipulate subcortical dopaminergic reward processing, with the potential to reduce anhedonia and replicate therapeutic benefits of DBS in a more accessible form.

An additional consideration when optimizing effects of striatal stimulation is an individual’s psychological state during stimulation. Practices designed to enhance positive mood and reduce negative emotionality, such as meditation, can potentiate stimulation effects(20) given that meditative practices tend to reduce stress and anxiety(21) and augment present-moment savoring(22). Recent reviews of non-invasive neuromodulation (e.g., transcranial magnetic stimulation, tACS) paired with meditation, indicate that neuromodulation appears to boost the effects of meditation itself; however, few studies have shown evidence of precise targeting of specific neural regions or networks, particularly those with subcortical involvement (for reviews, see 23, 24). Pairing excitatory VS TI with meditation may facilitate reward sensitivity by leveraging neural and psychological mechanisms.

Functional magnetic resonance imaging (fMRI) studies indicate that the VS is activated during reward tasks(25), and electroencephalography (EEG) studies probing reward sensitivity have characterized the reward positivity (RewP), an event-related potential (ERP) occurring approximately 250–350 ms following presentation of reward stimuli. It is typically measured as a difference wave between responses to rewarding and non-rewarding/punishing outcomes (ΔRewP), with rewarding stimuli eliciting a greater positive deflection following stimulus presentation(26). The RewP correlates with VS activation during fMRI(27, 28) and behavioral performance on reward tasks(29). Additionally, the RewP mimics patterns of subcortical dopaminergic firing related to reward characteristics, such as increased amplitude during uncertain rewards and greater reward magnitude (30 for review) and attenuation when rewards are temporally distant from the agent’s action(31–33). Blunted RewP response is consistently observed in depressed populations(34–37), and longitudinal studies of at-risk adolescents who later develop depression(38, 39). Successful depression treatment response has been linked to the RewP(40) and smaller pre-treatment RewP predicted greater response to selective serotonin reuptake inhibitors or cognitive behavioral therapy(41, 42). In sum, the RewP is a relevant and modifiable marker of reward sensitivity and dysfunction.

In this study, we tested whether temporal interference (TI) stimulation targeting the bilateral ventral striatum (VS) during breath-focused meditation affects the reward positivity (RewP) and emotional state in a sample of healthy adults. We administered a reward-related (Doors) task during EEG before and after each stimulation. We used a crossover study design. All participants completed active (8 Hz envelope) and high frequency sham (0 Hz envelope) TI stimulation on separate days with single-blind randomization of stimulation order (active TI first or sham first). An 8 Hz frequency within the high theta/low alpha range was utilized given that oscillatory activity at ~8Hz appears to play a key role in reward-related neural dynamics, with high theta VS showing associations with reward learning in animal models(51), and midline theta and low-alpha rhythms linked with meditative engagement and related emotion enhancement(52–54). To assess VS targeting specificity, we also examined TI effects on the N200 (N2), an ERP more relevant to cognitive control/response inhibition(55) than reward. We hypothesized that active TI stimulation would potentiate the RewP, but not N2, and high-frequency sham TI would have a null effect on both markers. Secondarily, we were interested in corresponding TI-related changes in emotion during meditation sessions and hypothesized that active TI stimulation of the bilateral VS would increase positive emotion and reduce negative emotion during breath-focused meditation, and that these effects would be linked with RewP changes. Finally, we examined potential carryover effects of stimulation across visits.

## 2. Material and Methods

### 2.1. Participants and Procedures

Nineteen healthy adults between 18–50 years old [13 (62%) female; Mean age = 25.38 years; SD = 5.99 years] were recruited from the community and completed all study procedures with good data quality. Two additional participants had been recruited but were excluded from analyses [one due to drop out at their second visit (discomfort from the stimulation) and one due to outlier EEG data (7.6 SDs above the mean)]. Participants underwent screening for potential contraindications for MRI or TI, which included presence of metal implants or other MR-unsafe medical devices, claustrophobia, irremovable body jewelry, history of epilepsy or medications that may increase the risk of seizures or tinnitus. Participants with current or history of serious or unstable medical illnesses, sustained loss of consciousness, substance use disorder, psychotropic medications or psychiatric illness were excluded for data quality. The Emory University Institutional Review Board approved study procedures.

Participants completed three study visits. During the first visit they provided informed consent, completed study questionnaires, and underwent a structural MRI scan. Next, they completed two single-blind stimulation sessions with active and sham TI on separate dates (detailed below). Stimulation order (active TI or sham TI first) was counterbalanced using a randomized cross-over design. To assess blinding efficacy, participants received an open-ended questionnaire at the conclusion of each visit to report when they believed the stimulation was on or off and where they felt effects.

### 2.2. TI Protocol

We applied TI using two DS5 Isolated Bipolar Constant Current Stimulators (Digitimer, Hertfordshire, UK). A Keysight waveform generator (Keysight Technologies Inc., Santa Rosa, CA) created a stimulation signal delivered to each stimulator. To generate an active TI envelope of 8 Hz, we set frequencies to 5000 Hz (f1) for one stimulator and 5008 Hz (f2) for the second stimulator, resulting in a TI envelope frequency of 8 Hz (f1-f2 = Δf); we utilized high carrier frequencies (> than 1 kHz) and square waves, similar to prior research(56)(57). Amplitude for each pair was standardized at ±4mA (8mA peak-to-peak) resulting in estimated E-field nonzero average of 3.179 V/m for the VS target. E-field simulations were run using the Sim4Life (ZMT Zurich MedTech AG, Switzerland) platform for computational life science based upon each participant’s structural MRI data. Full details of the electromagnetic field computation methodology are detailed in Supplement. High frequency sham TI stimulation consisted of two envelope-free carriers, i.e. 5000 and 5000 Hz, at the same coordinates and amplitude as active TI. At the stimulation visits, Cartesian coordinates from the MRI-guided stimulations were used in tandem with a Brainsight TMS-MRI co-registration system (Rogue Research, Quebec, Canada) with a Polaris stereo camera.

During TI stimulation sessions, participants were instructed to mindfully focus their attention on their breath (i.e., breath-focused mindfulness meditation) for 15 minutes, similar to our current clinical trial(58). Stimulation lasted for the entire duration, and participants completed subjective ratings of their emotional experience at the midpoint (7.5 minutes) and end (15 minutes) of stimulation.

### 2.3. Structural MRI Image Acquisition for VS Targeting and EEG Recording

Structural MRI data were acquired on a Siemens 3-Tesla PRISMA scanner (Siemens AG, Munich, Germany) using a 32-channel head coil. Structural images were scanned using a single-shot, high-resolution magnetization-prepared rapid acquisition gradient-echo sequence (MPRAGE; repetition time/echo time = 1900/2.27ms; flip angle = 9; field of view=250×250mm; 192×1.0mm slices). EEG was recorded using an elastic cap with 32 actiCAP slim electrodes positioned in accordance with the 10/20 system using a ActiChamp Classic amplifier (Brain Products GmbH). Electrode Cz served as an online recording reference, with a ground electrode placed at FPz. Left (TP9) and right (TP10) mastoid electrodes were placed directly on the surface of the skin for later re-referencing. The EEG signal was digitized at 1000 Hz and impedance kept below 25 kΩ. Detailed information regarding TI head modeling, electromagnetic field computation, and electrode placement provided in Supplement.

### 2.4. Task Design

**Figure 1.**
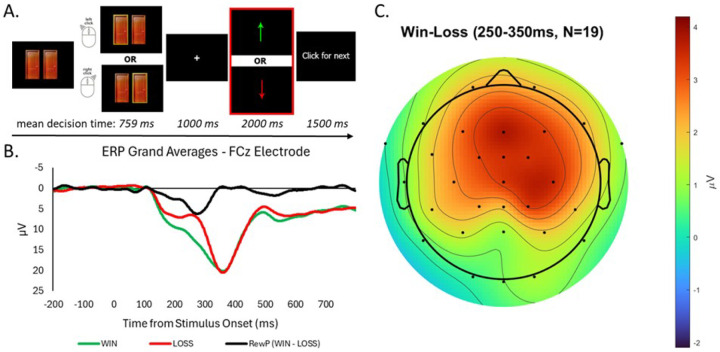
Doors Task. ERP Grand Averages, and Scalp Topography. **A.** Flow chart of the Doors task utilized to elicit the RewP based on ERP waveform averages of the outcome stimuli (highlighted in red). **B.** ERP averages of win trials, loss trials, and difference waves at the presentation of the outcome stimulus. **C.** Scalp topography of the win minus loss difference wave (RewP) from 250–350ms after reward outcome. Note: Channels FC6/T8 are not shown as they were often re-positioned at FCz as the reference during acquisition (varied depending on stimulation electrode placement).

We employed a widely-used task of reward responsivity, the Doors task (Fig. 1A) to probe VS-mediated reward function, similar to prior studies(59, 60). Participants viewed a fixation cross (500 ms) followed by an image of two identical doors, which remained until participants clicked either the left or right mouse button. Participants were told that they would either win or lose money on each trial and to guess which door would result in monetary gain. After another fixation cross (interstimulus interval: 1000 ms), feedback indicating monetary gain (green upward arrow) or loss (red downward arrow) was presented (2000 ms)(61). Participants were told they could either win $0.50 or lose $0.25 on each trial, with winnings accumulating. Finally, a fixation cross (intertrial interval) was presented for 1500 ms. Twenty win trials and 20 loss trials were presented in pseudorandom order, detailed further in Supplement.

### 2.5. EEG Processing

EEG data were analyzed using EEGLab, version 2024.2(62); processing details provided in Supplement. Feedback event-related potential epochs were averaged separately for win and loss trials as well as the Doors cue. RewP was quantified by contrasting the average signal between 250 and 350 ms at FCz following win and loss feedback as done previously(26, 35). For the Doors cue, we quantified N2 as the average signal between 200 and 350 ms at FCz following the Doors cue [Cue-N2(65)]. The Cue-N2 was extracted as a comparator ERP at the same location and similar time as the RewP(66). Given that windowed average for ERP responses may obscure the contributions of multiple components(67), we also extracted the peak positivity of RewP and peak negativity of Cue-N2 between 200–400 ms after stimulus onset for supplemental confirmatory analyses, consistent with prior recommendations(67, 68). Following data cleaning, one participant’s data was excluded from all analyses due to excessively noisy EEG data at their first stimulation visit. A final sample of *N* = 19 was available for all statistical analyses. Demographic and other descriptive information for this final sample provided in Table 1. Additional time frequency analysis on reward outcome was conducted and detailed in Supplement.

### 2.6. Statistical Analyses

Statistical analyses were conducted using the Statistical Package for the Social Sciences Version 29 (SPSS; [IBM, 2020]). To test our primary hypothesis, two mixed repeated-measures ANOVAs were conducted with dependent variables of RewP and Cue-N2 and independent variables of stimulation (active: 8 Hz; sham: 0 Hz), time (pre-stimulation, post-stimulation), and order (active-first, sham-first). We also tested the stimulation × time interaction (primary effect of interest) and the stimulation × time × order interaction (exploratory effect of interest). Secondary mixed repeated-measures ANOVAs were conducted with ERPs in response to Win and Loss outcomes separately to examine specificity of stimulation effects.

To test our second hypothesis, we conducted a multivariate ANOVA (MANOVA) with subjective experience ratings as dependent variables (happy, anxious, angry, numb, ability to regulate emotions, effortfulness of meditation). Independent variables were stimulation (active: 8 Hz; sham: 0 Hz), time (midpoint, endpoint of stimulation) and order (active-first, sham-first) with interactions of stimulation × time and stimulation × time × order. An α threshold of *p*< .05 indicated statistical significance.

To examine associations between changes in ERPs and subjective experience ratings, we conducted a cross-validated leave-one subject-out (LOSO) multivariate analysis using partial least squares (PLS) regression(69) in Matlab Statistics Toolbox(70) which is robust to collinearity(71). We conducted two PLS models for the active-sham TI difference in change in Win and Loss ERPs with the active-sham TI difference in change in a total of six ratings as predictors (happy, anxious, angry, numb, ability to regulate emotions, effortfulness of the meditation). ERPs were separated by Wins and Losses as difference scores between task conditions for behavioral and neural data are often less reliable for correlational analyses compared to scores from individual task conditions(72, 73). This also allowed us to assess whether changes in subjective ratings due to TI were more relevant to gains or losses. For each PLS model, permutation tests run with 10,000 iterations were used for statistical inference of cross-validated correlations with a permuted *p*< .05.

## 3. Results

### 3.1 Adverse reactions and blinding efficacy

Thirteen participants reported feeling stimulation during both sessions (active and sham). An additional 2 participants reported perceiving stimulation during the sham session only, and 2 other participants reported perceiving stimulation during the active session only. Two participants reported not perceiving stimulation during either session. Qualitatively, most participants indicated uncertainty regarding when stimulation was on or off. Collectively, responses provide support for blinding efficacy (Table S6 details full responses).

Two participants reported minor adverse reactions. One participant reported feeling uncomfortable during stimulation and was withdrawn from the study. Another participant reported mild headache following the TI session but noted a personal history of headache susceptibility. Their data was retained in analyses.

### 3.2. Effects of TI on the RewP vs Cue-N2 ERPs

**Figure 2.**
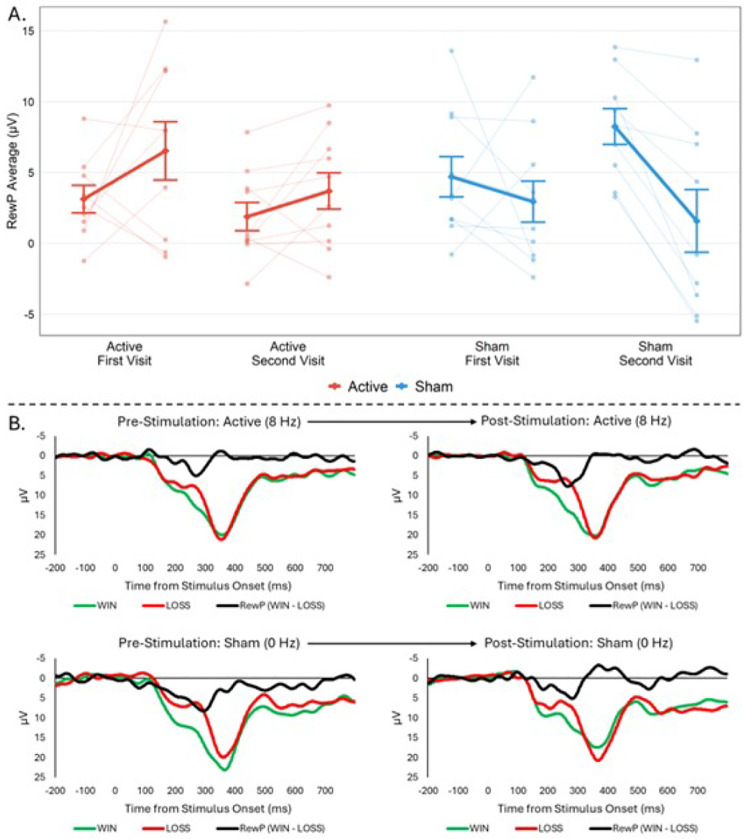
Group and Individual Average Changes in the RewP. **A.** RewP group averages and individual subject averages at 250–350ms post-reward outcome for the Win-Loss difference between pre- and post-active and sham stimulation separated by visit. Error bars indicate +/− 1 standard error of the mean. There was a significant stimulation × time interaction on the RewP at *p*<.001 and a significant stimulation × time × order interaction at p=.. **B.** ERP averages of win trials (RewP-win), loss trials (RewP-loss), and difference waves (ΔRewP) at the presentation of the outcome stimulus, split by stimulation (Active, Sham) and timepoint (pre-stimulation, post-stimulation). Analysis reveals a potentiation of the RewP post-active simulation and attenuation of the RewP post-sham stimulation.

ERP grand averages (across all four Doors task EEG runs) for Win and Loss trials and the RewP (Wins-Losses) are depicted in Fig. 1B. Estimated marginal means for each stimulation condition and time point for RewP and Cue-N2 ERPs and partial ANOVA results provided in Table 2; full ANOVA results in Table S1. For our primary hypothesis and effect of interest, ANOVA results showed a significant stimulation × time interaction on the RewP (*p*<.001; *η*_*p*_^*2*^ = .61); active TI led to a pre- to post-stimulation RewP increase whereas sham TI showed a RewP decrease (Fig. 2). For Cue-N2, there were no significant main effects of stimulation or time, or any interactions (*p*’s>.18). Secondary ANOVAs separating Win and Loss trials showed a significant stimulation × time interaction for Win trials (*p*=.004; *η*_*p*_^*2*^ = .39) but not Loss trials (*p*=.24). Win ERP increased for active TI and decreased for sham TI.

### 3.3 Effects of TI on RewP and Cue-N2 across visits.

**Figure 3.**
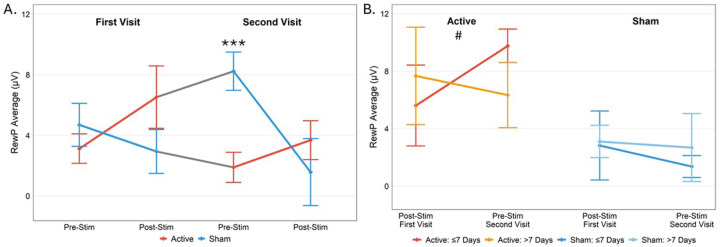
Effect of active vs sham stimulation on RewP across sessions, separated by order. **A.** Participants who received active TI (in red) during the first visit showed potentiation of the RewP during the second visit relative to individuals who received sham stimulation (in blue) first, indicating potential carryover effects between sessions (in gray). ***indicates significant pairwise difference at *p*<.001. **B.** Participants who received active TI first and had ≤ 7 days between stimulation visits (in red) showed a positive carry-over effect on their RewP across sessions while those who had > 7 days between stimulation visits (in orange) did not. This was not observed for sham stimulation with ≤ 7 days between visits (in blue) and those who had > 7 days between visits (in light blue), ^#^indicates marginally significant difference at a one-tailed *p*=.082.

A significant stimulation × time × order interaction (*p*=.024; *η*_*p*_^*2*^ = .27) emerged, showing more pronounced pre/post effects of both active and sham TI at stimulation visit 2, seemingly driven by a carryover effect from stimulation visit 1 to 2. This was evidenced by a pairwise difference in RewP during the pre-stimulation Doors task at visit 2 between the active-first and sham-first TI groups (*p* < .001; Fig. 3A). Follow-up exploratory analyses were conducted to examine impact of the number of days between visits on the carryover effect by dividing participants into two groups: those with ≤ 7 days between stimulation visits and those with > 7 days between stimulation visits. In the active-first TI group, there was a non-significant difference (one-tailed *p*=.082; *η*_*p*_^*2*^ = .26; Fig. 3B); those with ≤ 7 days between stimulation visits (*n* = 5; mean = 4.4 days) showed a positive carryover effect whereas those with > 7 days between stimulation visits did not (*n* = 4; mean = 22.5 days). No such effect was observed in the sham-first TI group (one-tailed *p*=.39) when comparing those with ≤ 7 days between visits (*n* = 6; mean = 5.3 days) to those with > 7 days between visits (*n* = 4; mean = 26.5 days).

### 3.4. Effects of TI on Subjective Experience and Multivariate Associations with ERPs

Estimated marginal means for each stimulation condition and time point for subjective experience ratings are shown in Table S2. MANOVA showed a significant stimulation × time interaction (*p*=.041; *η*_*p*_^*2*^ = .62); full results in Tables S3–4. Follow-up pairwise comparison for individual ratings of anxiety was significant (*p*=.044; *η*_*p*_^*2*^ = .22). Active TI showed a decrease in anxiety from the midpoint to endpoint of stimulation whereas sham TI showed an anxiety increase (Table S2).

PLS regression results showed that changes in subjective experience ratings due to active TI were significantly predictive of change in the Win ERP (cross-validated *r* = 0.526, permuted *p* = 0.016) but not Loss ERP (cross-validated *r*=−0.014, permuted *p* = 0.367). Full PLS results shown in Table S5. To directly compare level of prediction of changes in Win and Loss ERPs by changes in affective ratings, we conducted permutation testing on the difference between each PLS regression. The association with Win ERPs was significantly stronger than the association with Loss ERPs (cross-validated *r* = 0.540, permuted *p* = 0.036).

## 4. Discussion

We used a single-blind, cross-over design to examine neural and emotional changes produced by temporal interference (TI) stimulation targeting the ventral striatum (VS) during breath-focused mindfulness meditation. We used pre- and post-stimulation EEG to assess change in the amplitude of the RewP as an index of VS target engagement and examined changes in emotion ratings across sessions. We found that, unlike sham stimulation, TI increased amplitudes of a reward-related ERP, the RewP, but no significant changes emerged in an unrelated ERP, the Cue-N2, suggesting specificity of engagement during reward feedback. Additionally, we found some evidence of TI-related changes in neural function over a span of days; among participants who received TI before sham, this increase in RewP amplitude was sustained at the beginning of the second (sham) visit. Regarding emotion ratings, active TI—but not sham—stimulation produced changes during meditation sessions, specifically, a decrease in anxiety. This change during active TI stimulation was associated with increased amplitude of the win-related RewP. Collectively, these findings provide promising evidence that when targeting the VS, TI modulates EEG indices of reward processing, supporting prior research demonstrating TI’s capability to focally influence brain activity and related emotions(4–6).

To our knowledge, this is the first study to demonstrate that TI to the VS can potentiate the RewP, a candidate biomarker for anhedonic depression thought to assay reward sensitivity(78). In response to active TI, but not sham, an overall increase in RewP amplitude was observed, primarily driven by enhancement of the RewP to win outcomes. The lack of significant change in the RewP-loss response may reflect greater complexity in its neural origins, possibly involving a broader network, or may support the interpretation of prior principal component analyses, suggesting the RewP reflects a reward-specific signal, with loss trials marked by the absence of this positive deflection rather than a distinct negative component(79). It is also worth noting that although the RewP to win trials has demonstrated high internal consistency and test-retest reliability in adults, findings have been mixed for the loss-related RewP and the ΔRewP despite better reliability for the RewP-win signal(80–84). Further, we observed evidence of TI target specificity, as ERP changes were found in the RewP but not the Cue-N2, which marks a relatively earlier stage of cognitive processing (i.e., attention) whereas the RewP is evoked during elaborative, evaluative stages of processing [i.e., reward appraisal(85)].

A similar pattern of selective effects to RewP win outcomes has been observed following multiple doses of amphetamine relative to placebo(86), suggesting that effects of TI stimulation to VS may partly reflect an increase in striatal dopamine levels. Similarly, prior work by our group using intermittent theta burst stimulation (iTBS) to indirectly stimulate the striatum via targeting of a ventral prefrontal cortex region with high VS connectivity has also shown the ability to potentiate the RewP, with some evidence for greater effects on win trials(87). Importantly, the current work shows significantly larger effect sizes as compared to the iTBS study, as well as temporal specificity to the RewP versus the N2. Although we did not directly measure effects of TI on neural function with a spatially resolved method (e.g. fMRI), the specificity of effects on the RewP vs N2 suggest some specificity of TI effects. Taken together, these results suggest that TI stimulation may be a viable alternative to neuromodulation treatments that target subcortical areas via cortical-subcortical connectivity, such as the SAINT protocol for major depressive disorder treatment(88).

Consistent with our hypotheses, TI stimulation, but not sham, also influenced changes in self-reported emotion during meditation. Participants reported significant emotion changes, with the most robust effect being a decrease in anxiety; this change in anxiety correlated with the magnitude of RewP-win potentiation. Although these effects were modest (*p* = .04), they are noteworthy given our relatively small sample size and provide preliminary support for the potential of TI to enhance mood in the context of meditation. Findings complement existing studies pairing non-invasive neuromodulation with meditation, suggesting that TI could similarly enhance mindfulness-based interventions, which have previously shown emotional benefits when combined with other forms of transcranial electrical stimulation(89, 90).

Finally, supplemental time-frequency analysis was conducted to assess whether EEG spectral changes aligned with the TI stimulation envelope frequency of 8 Hz during reward outcome processing. While no significant differences were observed in the 7–9 Hz bands during the RewP window (250–350 ms), a notable increase in 8 Hz event-related spectral power (ERSP) emerged just prior, between 150–200 ms post-stimulus. Although further source localization and component analysis are necessary to determine the significance of this finding, the timing and frequency suggest a potential link to the P2a component—a motivationally relevant ERP known to be sensitive to outcome valence and associated with dopaminergic reward processing(91, 92).

## 5. Limitations

We acknowledge several important study limitations. First, although the RewP to win trials has demonstrated high internal consistency and test-retest reliability in adults, findings have been mixed for the loss-related RewP and the ΔRewP despite better reliability for the RewP-win signal(80–84). To address this concern, we employed a within-session design, comparing pre- and post-stimulation measures within a single visit, which reduces influence of between-session variability and allows for analysis of change-over-time effects. Second, although simulations were used to optimize field intensity at the VS, other nearby structures were also likely affected due to spatial extent of the TI envelope. However, the complexity of the surrounding neural architecture makes it difficult to fully isolate the target and interpret these potential off-target effects. Studies using multi-polar TI are growing (e.g.,(93)), and this application of TI may reduce the spatial spread of stimulation and improve focality (94). Third, 8 Hz stimulation is slightly higher than the 5Hz frequency that has been most widely used in TI studies (e.g. 4, 95, 96). However, exact boundaries between high-theta and low-alpha frequencies are a matter of debate, and use of 8 Hz was guided by prior EEG work in meditators(53, 97). Further investigation is needed to validate this frequency and compare its effects against other stimulation frequencies before making broader recommendations. Finally, although ERP analyses were adequately powered, our limited overall sample size likely reduced sensitivity for detecting more subtle emotion effects; replication in a large clinical sample is merited. Nonetheless, we observed large effects with a comparable sample size to other published TI studies (e.g. 56, 98).

## 6. Conclusions

To our knowledge, these findings provide the first evidence that TI to the VS during breath-focused mindfulness meditation modulates the amplitude of a reward-related neural marker, the RewP, with sustained effects over time. We also observed TI-specific effects on anxiety during meditation. Data showing selective and sustained neural changes both within and between visits may support the notion that TI induces neuroplasticity in a deep brain region that is inaccessible to most non-invasive neuromodulation methods. The lack of serious adverse events bolster previous findings indicating TI has similar safety recommendations as established methods, such as tACS, and good blinding efficacy(99). This work adds to the growing literature on TI and highlight the promise of this method as a tool for modulating reward circuitry, underscoring its potential as a non-invasive intervention for treating disorders of reward dysfunction.

## Supplementary Files

This is a list of supplementary files associated with this preprint. Click to download.

- TIRewPStriatumSupp9925.docx
